# Potential of Vitamin E Deficiency, Induced by Inhibition of α-Tocopherol Efflux, in Murine Malaria Infection

**DOI:** 10.3390/ijms20010064

**Published:** 2018-12-24

**Authors:** Hiroshi Suzuki, Aiko Kume, Maria Shirely Herbas

**Affiliations:** 1 Research Unit for Functional Genomics, National Research Center for Protozoan Diseases, Obihiro University of Agriculture and Veterinary Medicine, Nishi 2-13, Inada, Obihiro 080-8555, Japan; kume.a@obihiro.ac.jp (A.K.); herbas86@yahoo.com (M.S.H.); 2 The United Graduate School of Veterinary Sciences, Gifu University, Gifu 501-1193, Japan

**Keywords:** Vitamin E, α-tocopherol, α-tocopherol transfer protein (α-TTP), oxidative stress, probucol, chloroquine, artemisinin-based combination therapy (ACT)

## Abstract

Although epidemiological and experimental studies have suggested beneficial effects of vitamin E deficiency on malaria infection, it has not been clinically applicable for the treatment of malaria owing to the significant content of vitamin E in our daily food. However, since α-tocopherol transfer protein (α-TTP) has been shown to be a determinant of vitamin E level in circulation, manipulation of α-tocopherol levels by α-TTP inhibition was considered as a potential therapeutic strategy for malaria. Knockout studies in mice indicated that inhibition of α-TTP confers resistance against malaria infections in murines, accompanied by oxidative stress-induced DNA damage in the parasite, arising from vitamin E deficiency. Combination therapy with chloroquine and α-TTP inhibition significantly improved the survival rates in murines with malaria. Thus, clinical application of α-tocopherol deficiency could be possible, provided that α-tocopherol concentration in circulation is reduced. Probucol, a recently found drug, induced α-tocopherol deficiency in circulation and was effective against murine malaria. Currently, treatment of malaria relies on the artemisinin-based combination therapy (ACT); however, when mice infected with malarial parasites were treated with probucol and dihydroartemisinin, the beneficial effect of ACT was pronounced. Protective effects of vitamin E deficiency might be extended to manage other parasites in future.

## 1. Introduction

Nutrition might be one of the numerous biological, immunological, and ecological factors that influence the adaptation of human population to *P. falciparum* malaria. Vitamin E, a fat-soluble nutrient found in food, includes four tocopherols and four tocotrienols, designated as α, β, γ, and δ. Alpha-tocopherol is essential, owing to its highest biological antioxidant activity in many species. Its deficiency may cause abortion, neurological dysfunction, myopathies, and diminished erythrocyte lifespan [1]. 

The exacerbative effects seen in cerebral malaria, following re-feeding of famine victims with grain, were possibly due to the high vitamin E content of the grain [2]. Since vitamin E content of milk is low, that is one of the adaptive aspect of high-milk diets of many African pastoral population. Tropical palm and coconut oils also have lower vitamin E content. Therefore, consumption of these oils may provide some antimalarial protection, relative to that of corn, soybean, and other vegetable oils that have higher levels of vitamin E [3]. In fact, Modiano et al. observed much lower *P. falciparum* infection rates, at all ages, among a nomadic pastoralist population compared to that in two other sedentary farming populations in Burkina Faso [4,5]. Therefore, these clinical observations indicate that lower vitamin E levels may have a beneficial effect on the course of malaria infection in humans [6]. In addition, evidence from animal studies suggests that vitamin E deficiency has a beneficial antimalarial effect on the course of malaria infection [7,8,9,10,11,12,13]. However, even if it might be possible to utilize vitamin E deficiency for the prevention and/or treatment of infectious diseases, it would be difficult to actually lower vitamin E in circulation via nutritional manipulation, since the majority of daily foods contain significant amount of vitamin E. Thus, α-tocopherol deficiency was believed to be inapplicable in clinical therapy of malaria.

**Figure 1:**
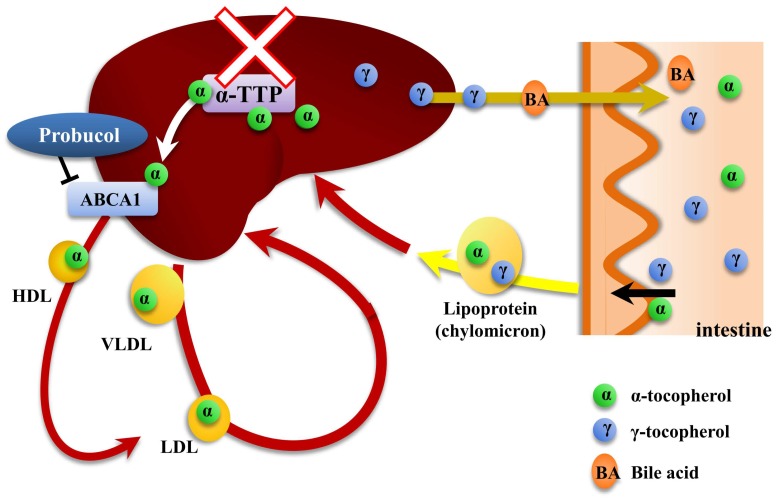
Possible induction of vitamin E deficiency by the inhibition of α-tocopherol efflux from liver cells. Both α- and γ-tocopherols are absorbed similarly from the intestine and transported by chylomicrons in plasma. Following the uptake of chylomicrons by the liver, only α-tocopherol is preferentially transported and localized to plasma membrane of liver cells by α-tocopherol transfer protein (α-TTP). It is subsequently incorporated into lipoproteins, resulting in recirculation of this tocopherol in the body. On the other hand, γ-tocopherol is taken up by the liver and eventually secreted into bile. ATP-binding cassette transporter A1 (ABCA1) participates directly in the transport of α-tocopherol from liver cells to Apo A-I, which is the major protein component of HDL. Thus, α-TTP or ABCA1 might be suitable targets to induce vitamin E deficiency in circulation.

More recently, vitamin E homeostasis has been demonstrated to be regulated by the α-tocopherol transfer protein (α-TTP) in liver [14,15]. Both α- and γ-tocopherols are similarly absorbed from the intestine and transported by chylomicrons in plasma. Following uptake by the liver, only α-tocopherol is preferentially incorporated into VLDL and HDL by cytosolic α-TTP, resulting in recirculation of this tocopherol in the body. The other isoforms are metabolized by microsomal P450 or excreted into the bile. Efflux of α-tocopherol from liver cells into HDL is mediated by ATP-binding cassette transporter A1 (ABCA1) [16,17]. Thus, α-TTP plays a major role in maintaining adequate levels of α-tocopherol in plasma [15]. Considering the manipulation of α-tocopherol levels by α-TTP inhibition, we were inspired to revisit the impact of serum vitamin E levels on the severity of malaria infection. As expected, the established α-TTP gene knockout mouse showed undetectable levels of vitamin E in circulation [14]. Moreover, ABCA1, a downstream molecule for α-tocopherol efflux from liver cells into circulation, might also be a target to induce vitamin E deficiency in circulation [18] (Figure 1).

## 2. Effect of Vitamin E Deficiency, due to α-TTP Gene Disruption, on Murine Malaria Infection

### 2.1. Integrity of RBC Membrane in α-TTP Knockout Mice

Diet-induced vitamin E deficiency is known to be harmful for red blood cell (RBC) integrity [19]. When RBCs from mice, fed on a vitamin E-deficient diet or normal diet, were compared with those from α-TTP knockout mice, no significant difference was observed in RBC counts, hematocrit values, and hemoglobin concentrations between the normal diet-fed mice and α-TTP knockout mice [20]. In contrast, the hematological parameters in mice fed on vitamin E-deficient diet were significantly decreased relative to that in normal diet-fed mice and in α-TTP knockout mice. Signs of anemia, such as a pale color of the tail and limbs, were evident in the vitamin E-deficient diet-fed mice while α-TTP knockout and normal diet-fed mice did not show any sign of anemia. Osmotic fragility, oxidation sensitivity, and hemolysis susceptibility of the RBCs from mice on vitamin E-deficient diet were higher than in those from normal diet-fed and α-TTP knockout mice. There was no significant difference in these parameters between the RBCs from normal diet-fed mice and α-TTP knockout mice (our unpublished observation). Therefore, the capacity of RBCs from the knockout mice seems to be maintained in a stable status. However, vitamin E deficiency, through dietary manipulation, has a dramatic impact on the stability of RBC. α-TTP gene inhibition is not harmful for RBC stability, even though vitamin E concentration in circulation is undetectable. Alpha-TTP knockout mice might possess the capacity to maintain adequate concentration of vitamin E in the RBC membranes, in order to be able to prevent hemolysis, and thus avoid anemia. Either α-TTP knockout mice absorb vitamin E from their diet in order to maintain acceptable levels of vitamin E in the RBCs, as demonstrated in patients with familial isolated vitamin E deficiency [21], or the missing vitamin E content is compensated by other anti-oxidants such as ascorbate and β-carotene. In fact, the concentration of reduced glutathione, an indicator of oxidative status, in the liver of α-TTP knockout mice was similar to that seen in wild type mice [22]. 

Both malarial parasites and RBC membranes of vitamin E deficient diet-fed mice are highly susceptible to peroxidation due to the production of reactive oxygen species, since the nature of dietary fat alters the lipid composition of RBC membranes and malarial parasites cannot biosynthesize their own fatty acids [23]. 

### 2.2. Inhibition of α-TTP Confers Resistance to Malaria Infection

When α-TTP knockout mice, with C57BL/6J genetic background, were infected with a lethal dose of *Plasmodium berghei* NK65-infected RBCs (4 *×* 10^5^), their survival was significantly extended compared to that in wild type mice [22]. Survival was longer and parasitemia lower in the α-TTP knockout mice than in the naturally *P. berghei* NK65-resistant BALB/c strain of mice [22]. The effect of α-TTP gene disruption was much more remarkable for a *P. yoelii* 17XL infection. Infection with 4 × 10^4^ *P. yoelii* 17XL-infected RBCs killed all the wild type mice by day 9 after infection, whereas all the knockout mice survived with the disappearance of parasitemia [22]. Decreased vitamin E concentration seems to generate an environment of higher oxidative stress in the infected parasites in α-TTP knockout mice. Comet assay and immunofluorescence staining with 8-OHdG antibody revealed oxidative DNA damage in parasites recovered from α-TTP knockout mice [22]. In the absence of infection, there was no significant difference in the percentages of mature and immature RBCs (reticulocyte) within the total RBC pool, and in the mRNA expression levels of erythropoietin (EPO) between α-TTP knockout and wild type mice [22,24]. However, the percentage of infected reticulocytes in the total pool of infected RBCs was significantly higher in α-TTP knockout mice than in wild type mice, throughout the *P. berghei* NK65 infection [22]. In the knockout mice, parasites prefer to invade the newly produced cells rather than mature RBCs, probably to evade the increased oxidative stress derived from vitamin E deficiency. These results indicate the increased possibility that virulence of parasites in α-TTP knockout mice would be affected owing to the oxidative stress-derived DNA damage in parasite. 

Interestingly, when α-TTP knockout or wild type mice were inoculated with *P. berghei* NK65 that had been recovered from previously infected α-TTP knockout mice, both α-TTP knockout and wild type mice survived significantly longer than these infected with parasites from wild type mice [22]. Thus, parasites recovered from α-TTP knockout mice were less virulent than those recovered from the wild type mice. Virulence of the parasites existing in α-tocopherol-deficient environment had decreased during the time it spent within the host.

There is a mouse model that closely mimics the features of human cerebral malaria [25], including the intravascular sequestration of macrophages and monocytes and the breakdown of blood–brain barrier permeability. When C57BL/6J mice were infected with 4 × 10^4^ *P. berghei* ANKA-infected RBCs, they exhibited neurological symptoms including convulsions, paralysis of the limbs, and rolling over, prior to the onset of death. However, none of these symptoms was observed in α-TTP knockout mice infected with *P. berghei* ANKA [26]. The breakdown of blood–brain barrier could be detected by an intravenous Evans blue injection in wild type mice infected with *P. berghei* ANKA. While the brains of α-TTP knockout mice did not show any brain capillary leakage throughout the course of infection [26], brain sections from the wild type mice showed severe lesions, such as hemorrhage, disruption of the vessel wall, enhancement of the perivascular space, and intravascular accumulation of mononuclear cells, prior to the onset of death. In contrast, the brain sections of α-TTP knockout mice did not exhibit vascular abnormalities throughout the course of infection, despite parasitemia in the knockout mice reaching levels that were sufficient for the development of cerebral malaria in wild type mice [26]. When mRNA expression levels of various adhesion molecules, such as vascular cellular adhesion molecule (VCAM), intracellular adhesion molecule (ICAM), lymphocyte function associated antigen (LFA-1), and glial fibrillary acidic protein (GFAP) were checked in the brains of α-TTP knockout and wild type mice infected with *P. berghei* ANKA, the expressions of ICAM, LFA-1, and GFAP were found to be significantly increased in wild type mice after infection, whereas they were not so during the acute phase of infection in α-TTP knockout mice [26]. Since ICAM-deficient mice, infected with *P. berghei* ANKA, also failed to develop cerebral malaria, even though parasitemia levels were similar to those observed in susceptible wild type mice [27], it is likely that the parasitemia level, by itself, has no critical influence on the outcome of cerebral malaria.

Alpha-TTP knockout mice, infested by 10–20 mosquitos infected with *P. berghei* ANKA, displayed survival rates similar to those of the α-TTP knockout mice intraperitoneally injected with infected RBCs [26]. Thus, α-TTP disruption may not influence the sporozoite stage of parasites in the liver (Pre-erythrocytic stage). 

When the α-TTP knockout mice were fed an excess amount of α-tocopherol-supplemented diet (600 mg/kg), their survival curves and parasitemia after *P. berghei* NK65 infection were similar to those of wild type mice fed a standard diet [22]. Similarly, the brains of α-TTP knockout mice, fed an α-tocopherol-supplemented diet, showed blood–brain barrier breakdown after *P. berghei* ANKA infection [26]. Thus, α-TTP knockout mice acquired resistance to malaria infection owing to vitamin E deficiency, not by α-TTP gene disruption itself.

### 2.3. Expression Levels of Anti-Oxidative Stress Enzyme in the Infected Parasites

Complete absence of vitamin E in the host circulation system would lead to an oxidative stress environment that would require parasites to efficiently use their inherent antioxidant system for survival [28,29,30]. Indeed, antioxidant activity was found to be enhanced in these parasites. Monitoring the mRNA expression of antioxidative stress enzymes, such as glutaredoxin (Grx), γ-glutamyl transferase (γ-GCS), 2-Cys peroxiredoxin (2-Cys Prx), and thioredoxin reductase (TrxR) of *P. berghei* NK65 parasites in α-TTP knockout mice, showed that, after the initial higher expression levels of Grx, and γ-GCS, transcription of these genes dropped to similar levels as in the parasites infecting the wild type mice [22]. This might be because the parasites in the knockout mice had adapted to the oxidative environment [31]. Inhibition of α-TTP gene activity leads to an inhospitable environment for parasites residing within the host. 

### 2.4. Cytokine Responses in α-TTP Knockout Mice

The mRNA expression of IL-10, INF-γ, and TNF-α in liver, kidney, and spleen of α-TTP knockout mice was similar to that in wild type mice throughout *P. yoelii* 17XL infection [32]. In a cerebral malaria model, the expression levels of IL-10, INF-γ, TNF-α, and IL-1β were significantly increased in the brains of C57BL/6J mice after *P. berghei* ANKA infection [26]. However, mRNA expressions of IL-10, INF-γ, TNF-α, and IL-1β were not increased during the acute phase of *P. berghei* ANKA infection in the brains of α-TTP knockout mice. Interestingly, despite the lack of symptoms of cerebral malaria in α-TTP knockout mice, mRNA levels of these cytokines were significantly up-regulated later on. In addition, mRNA expression levels of TNF-α and INF-γ in the liver and spleen were similarly up-regulated in both *P. berghei* ANKA-infected α-TTP knockout and wild type mice [26]. These results suggest that the inflammatory immune response of the knockout mice is not altered by α-TTP gene disruption. 

### 2.5. Enhancement of the Acquired Immune Response by α-TTP Inhibition

Non-lethal Plasmodium strains or genetically modified parasites have been reported to be capable of protecting or suppressing pathogenesis subsequent to lethal infections in mice [33,34,35,36]. *P. yoelii* 17XL, *P. berghei* NK65, and *P. berghei* ANKA infections were shown to be lethal in C57BL/6J strain of mouse; however, *P. yoelii* 17XL, among them, was non-lethal in α-TTP knockout mice with a C57BL/6J genetic background [22,26]. Parasitemia, in wild type mice infected with *P. yoelii* 17XL, increased dramatically from day 5 after the infection, and 100% death was observed by 2 weeks thereafter. In contrast, in α-TTP knockout mice, parasitemia was undetectable and survival rate was 100% throughout the observation period [22,32]. Due to the ability of α-TTP knockout mice to control *P. yoelii* 17XL infection, it was postulated that the slower proliferation of parasite may allow them to mount an efficient immune response in the animals. Interestingly, when α-TTP knockout mice were infected with *P. yoelii* 17XL, and subsequently challenged with *P. berghei* NK65 on day 15 after the initial infection, the knockout mice showed 100% survival with extremely low levels of parasitemia [32]. However, when α-TTP knockout mice were immunized with fixed *P. yoelii* 17XL parasites, no protective effect was seen. Thus, live parasites or live parasite-derived factors seem to be responsible for mounting an adequate and robust immune response. For antibody response, the production of IgG and IgG2c in the double infected knockout mouse was clearly activated after the secondary infection. This response was significantly higher when the *P. yoelii* 17XL crude antigen was used, rather than the *P. berghei* NK65 crude antigen [32]. These results indicate that α-TTP knockout mice, infected with *P. yoelii* 17XL, developed cross-immunity against *P. berghei* NK65 challenge. 

### 2.6. Expression of Erythropoietic Cytokines in α-TTP Knockout Mice with Malaria Infection

Anemia is the most general complication of malaria infection, especially in young children and pregnant women [37]. Although the precise mechanism of anemia, in relation to malaria, remains unclear, a stimulated rupture of infected and uninfected RBCs, along with dis-balanced e pro- and anti-inflammatory cytokine response of the host, and insufficient erythropoiesis [37,38] have been proposed in this context. EPO is an essential erythropoietic cytokine, produced mainly in the kidney of an adult, although its production occurs in the brain and liver at fetal stage [39]. Impaired EPO production in the kidney has been suggested to induce the production in alternative organs such as the liver and brain, which likely compensates for this deficiency [39]. Hypoxia triggers EPO production in the kidney. By binding to its receptors (EPOR) expressed on erythroid progenitors in bone marrow, erythroid progenitors are stimulated, and increased number of reticulocytes are released into the bloodstream [40,41]. Role of EPO during malarial anemia still remains controversial. Some studies agree with the fact that EPO production is inadequate under such circumstances [1,42] while others indicate that an exacerbated production of EPO might inhibit erythropoiesis [43]. 

The expression levels of EPO and reticulocyte count were not significantly different between the α-TTP knockout and wild type mice before infection. When these mice were infected with 4 × 10^4^ *P. berghei* NK65-infected RBCs, the hematological parameters such as RBC counts, hematocrit values, and hemoglobin concentrations slightly decreased with low levels of parasitemia in α-TTP knockout mice. In contrast, wild type mice exhibited significant reduction in RBC counts, hematocrit values, and hemoglobin concentrations, with higher level of parasitemia. Upon infection, the mRNA expression of EPO in the kidney was significantly increased in both α-TTP knockout and wild type mice compared to the pre-infection levels. Although significant enhancement of EPO expression was detected in the kidney of wild type mice with infection, number of reticulocytes was significantly decreased, suggesting a possible impairment of EPO biological activity [24]. In addition, EPO expressions were detected in the liver and spleen with the progression of anemia, suggesting a possible compensatory mechanism to accelerate erythropoiesis [24]. Nevertheless, role of the liver and spleen, with regard to EPO production in malaria infection, remains to be clarified in humans. Interestingly, the mRNA expression levels of EPOR was dramatically suppressed in the bone marrow at early stage of infection, independent of the severity of anemia, EPO expression, and parasitemia levels in both α-TTP knockout and wild type mice [24]. EPOR suppression at less than half of the pre-infection levels was reflected in the number of reticulocytes in wild type mice. On the other hand, α-TTP knockout mice showed enhanced number of reticulocytes after infection. Bone marrow activity might be partially impaired, and an effective compensatory mechanism by the liver and spleen might occur in α-TTP knockout mice after infection [24]. It has also been reported that mice infected with *P. yoelii* could have a potential to induce hepatic erythropoiesis to avoid anemia [44]. Since not all patients infected with Plasmodium suffer from anemia, a compensatory mechanism by other organs might play a key role in the outcome of anemia during malaria infection, in humans as well as in α-TTP knockout mice. 

Malarial parasites digest host hemoglobin, and consequently, it releases free heme. Free heme is further converted into an insoluble form called hemozoin. Hemozoin induces the release of macrophage migration inhibitory factor (MIF) and other cytokines from monocytes and macrophages [43]. Prominent expression of MIF in plasma, bone marrow, and spleen suggests that it may mediate erythropoietic suppression [45]. Bone marrow suppression, owing to MIF expression, might be attributed to a sustained effect of malarial attack or to the persistence of parasites that are undetectable by routine microscopy [46]. When α-TTP knockout and wild type mice were infected with *P. berghei* NK65, the mRNA expression of MIF increased dramatically in the bone marrow and spleen during the peak of parasitemia in wild type mice, but not in α-TTP knockout mice [24], hence suggesting that hemozoin might be a key factor in the inhibition of erythropoiesis, as described previously [47]. 

The role of spleen during malarial anemia has also been well documented [48]. In *P. berghei* NK65 infection, levels of parasitemia were significantly different between α-TTP knockout and wild type mice while the increase in spleen index (spleen weight/body weight) was similar between them [24]. Histological analysis revealed that an enlarged spleen in both α-TTP knockout and wild type mice, infected with *P. berghei* NK65, was caused due to the extension of white pulp with increase of B cell population. However, proliferation of erythroblasts was augmented in the red pulp of wild type mice, but not in the α-TTP knockout mice (our unpublished observation). In α-TTP knockout mice, EPO production is triggered in organs such as the liver and spleen, besides the kidney, whereas EPOR expression is inhibited in the bone marrow; however, other organs, such as spleen, might compensate for this function. 

### 2.7. A Combination Therapy with Chloroquine Administration and α-TTP Inhibition

When wild type mice were infected with 4 × 10^5^ *P. berghei* NK65-infected RBCs, and treated with 5 mg/kg of chloroquine on day 0, 1, and 2 after the infection, their survival was significantly improved while all of them eventually succumbed to malarial parasites by day 23 after the infection. In contrast, similar parasite inoculation and chloroquine treatment of α-TTP knockout mice resulted in 100% survival with undetectable levels of parasitemia [22]. Combination of chloroquine administration and α-TTP inhibition could potentially be used as a promising treatment for malaria infection. Since α-TTP inhibition led to DNA damage in the parasite, sufficient to inhibit proliferation, combined therapy with chloroquine seems to be useful as a new strategy for treatment of malaria.

## 3. Effect of Probucol-Induced Vitamin E Deficiency on Murine Malaria Infection

### 3.1. Reduction in α-Tocopherol Concentration after Treatment with Probucol

Based on the findings from infection experiments in α-TTP knockout mice, the potential of α-TTP inhibition as a therapeutic strategy lies in the ability to chemically inhibit the protein. In humans, an α-tocopherol-specific binding pocket in α-TTP has been revealed by crystallography, and is considered to be responsible for homeostasis of vitamin E [49]. On the other hand, clinical application of α-tocopherol deficiency would be possible, if a drug, capable of reducing α-tocopherol concentration, would be identified. It has been reported that treatment of hypercholesterolemic patients with 0.5 g probucol twice a day reduces serum vitamin E concentration by 14% [50]. In animal studies, dietary supplementation with 0.5% (*w*/*w*) probucol decreased plasma concentrations of vitamin E in LDL receptor knockout mice [51] while, supplementation with 1% (*w*/*w*) probucol decreased the concentration of α-tocopherol in the aortic arch and descending aorta of ApoE knockout mice [52]. Treatment with 1% (*w*/*w*) probucol for 2 weeks reduced plasma concentration of α-tocopherol by 10% in wild type mice [17,53].

Probucol, 4,4′-[(1-methylehylidene)bis(thio)]bis [2,6-bis(1,1-dimethylethyl) phenol], is a drug used for the treatment of hyperlipidemias [54], since it inactivates ABCA1-mediated cholesterol efflux [55,56]. It may be considered that inhibition of ABCA1, a downstream molecule for α-tocopherol efflux from liver cells [17,18], induces α-tocopherol deficiency in circulation.

Effect of dietary supplementation with 1% (*w*/*w*) probucol on the α-tocopherol concentration in mice is dramatic. Probucol reduced the plasma α-tocopherol concentration to 25% and 9% of the control levels after 1 day and 2 weeks of treatment, respectively. It decreased both plasma cholesterol and α-tocopherol concentrations after treatment with 1% (*w*/*w*) probucol, whereas the reduction in α-tocopherol levels was larger than that in plasma cholesterol levels [53]. Furthermore, the concentration of α-tocopherol in erythrocytes did not decrease after probucol treatment [53]. Thus, probucol does not reduce α-tocopherol levels of plasma by solely reducing plasma lipoproteins, which are the main reservoirs of α-tocopherol; rather, it selectively inhibits ABCA1, leading to α-tocopherol reduction.

Lowering of plasma α-tocopherol concentration by other anti-hyperlipidemia drugs with different actions such as ezetimibe [57], berberine [58], and cholestyramine [59] has been proven to be weaker than by probucol [60].

### 3.2. Effect of Probucol on the Murine Malaria Infection

When C57BL/6J mice were treated with 1% (*w*/*w*) probucol for 2 weeks and then infected with 2 × 10^4^ *P. yoelii* 17XL-infected RBCs, subsequently continuing with probucol treatment thereafter, 75% of the probucol-treated mice survived while all untreated control mice died by day 16 post-infection. Anemia was evident in both control and probucol-treated mice; however, the latter recovered from anemia [53]. Although signals of being 8-OHdG-positive were detected in the infected RBCs, taken from both 0.5% *w*/*w* probucol-treated and control mice infected with 4 × 10^4^ *P. yoelii* 17XL-infected RBCs, the mean fluorescence intensity in probucol-treated mice was significantly higher than that in control mice [61]. These results indicate that probucol influences malaria infection by oxidative stress via the induction of host vitamin E deficiency. 

When mice were infested with 5 mosquitos infected with *P. berghei* ANKA, the median survival of 1% (*w*/*w*) probucol-treated mice was on day 18 post-infection while it was on day 10 in untreated controls. Probucol-treated mice died with anemia and without clinical signs of cerebral malaria. However, all control mice died with both anemia and clinical signs of cerebral malaria [53].

Since plasma concentration of α-tocopherol significantly decreased on day 1 after 1% (*w*/*w*) probucol treatment, pre-treatment of probucol seems to be not so essential for malaria infection. Therefore, probucol treatment was initiated immediately after inoculation with 2 × 10^4^ *P. yoelii* 17XL-infected RBCs. In fact, survival rate was significantly extended in mice infected with *P. yoelii* 17XL and simultaneously treated with probucol [53]. 

## 4. A Combination Therapy with Dihydroartemisinin and Probucol

Although chloroquine had been considered to be the first-line drug for malaria treatment in the past, emergence of chloroquine-resistant Plasmodium strains have made malaria treatment difficult, especially in endemic areas [62,63,64]. Recently, artemisinin and its derivatives have become essential compounds for anti-malaria treatment and their rapid clinical parasitological responses are life-saving in cases of severe malaria [65]. However, the first artemisinin-resistant *P. falciparum* was reported in western Cambodia [66]. In 2014, drug efficacy studies had detected resistant *P. falciparum* in Cambodia, the Lao People’s Democratic Republic, Myanmar, Thailand, and Vietnam [67]. Drug combinations are effective in delaying or preventing the appearance of drug resistance [68]. Currently, treatment of malaria relies on the artemisinin-based combination therapy (ACT): a combined chemotherapy of artemisinin derivative with other anti-malarial drugs [69,70]. Despite the recent spread of artemisinin-resistant parasites [71], ACT is still highly efficacious. However, due to the slow-clearance of parasites in patients treated with ACT, more parasites are exposed to the partner drug, thereby increasing their risk of developing resistance to the partner drug [72]. Therefore, development of a novel method to enhance the effect of artemisinin would be required. 

The beneficial effect of combined treatment using dihydroartemisinin (DHA), an artemisinin derivative, and probucol was evident [53,61]. Mice were treated with 1% (*w*/*w*) probucol and infected with 2 × 10^4^ *P. yoelii* 17XL-infected RBCs, and then injected with 30 mg/kg of DHA on days 3, 4, and 5 after infection. Mice treated with probucol plus DHA showed significantly lower parasitemia than those treated only with DHA. No mortality was observed in either the combined therapy or single DHA treatment [53]. More recently, it has been shown that probucol is effective in ACT at much lower concentrations [61]. When mice were treated with 0.25% (*w*/*w*) probucol for 2 weeks, before infection with 4 × 10^4^ *P. yoelii* 17XL-infected RBCs, and then injected with 7.5 mg/kg of DHA on days 3, 4, and 5 after infection, their survival rate was 100%. Survival rate in the combined treatment was significantly higher than that in 7.5 mg/kg DHA alone (38%). Furthermore, combined treatment showed significantly higher hemoglobin concentration than single DHA treatment. Interestingly, the peak of parasitemia in mice treated with combined drugs (0.25% of probucol and 7.5 mg/kg of DHA) was equivalent to that in mice treated with DHA at a single dosage of 30 mg/kg [61]. These results indicate that prophylactic treatment with probucol enhances the effect of DHA in murine malaria infection. 

Artemisinin and its derivatives are considered to interact with heme/iron in the parasite food vacuole to generate free radicals that are toxic to the parasites [73,74]. However, unlike other oxidant drugs, artemisinin cannot be cyclically oxidized and reduced [75,76]. Therefore, one drug molecule can generate only one free radical [77]. Since anti-malarial effect of artemisinin was seen to be reduced by vitamin E treatment both in vitro [73] and in vivo [78], the beneficial effect of the combination treatment of DHA with probucol [61] might be attributed to the lowering of host plasma concentration of vitamin E by probucol, which prevented the elimination of free radicals generated by DHA. Such a strategy might be applicable to other parasites as well in future, such as trypanosoma [20].

## Abbreviations

α-TTP	α-Tocopherol transfer protein
ACT	Artemisinin-based Combination Therapy
VDL	Very Low Density Lipoprotein
LDL	Low Density Lipoprotein
ABCA1	ATP-binding cassette transporter A1
RBC	Red blood cell
EPO	Erythropoietin
8-OHdG	8-Hydroxy-2’-deoxyguanosine
VCAM	Vascular cellular adhesion molecule
ICAM	Intracellular adhesion molecule
LFA-1	Lymphocyte function associated antigen
GFAP	Glial fibrillary acidic protein
LFA-1	Lymphocyte function associated antigen
Grx	Glutaredoxin
γ-GCS	γ-glutam*y*l transferase
2-Cys Prx	2-Cys peroxiredoxin
TrxR	Thioredoxin reductase
EPOR	Erythropoietin receptor
MIF	Macrophage migration factor
DHA	Dihydroartemisinin
	
